# Cost-effectiveness analysis of microscopic observation drug susceptibility test versus Xpert MTB/Rif test for diagnosis of pulmonary tuberculosis in HIV patients in Uganda

**DOI:** 10.1186/s12913-016-1804-9

**Published:** 2016-10-10

**Authors:** Simon Walusimbi, Brendan Kwesiga, Rashmi Rodrigues, Melles Haile, Ayesha de Costa, Lennart Bogg, Achilles Katamba

**Affiliations:** 1 Department of Microbiology, Makerere University College of Health Sciences, Kampala, Uganda; 2 Department of Public Health Sciences, Karolinska Institute, Solna, Sweden; 3 HealthNet Consult, Kampala, Uganda; 4 Department of Microbiology, Public Health Agency of Sweden, Solna, Sweden; 5 Department of Medicine, Clinical Epidemiology Unit, Makerere University, College of Health Sciences, Kampala, Uganda; 6 School of Health, Care and social Welfare, Malardalen University, Vasteras, Sweden

**Keywords:** Cost-effectiveness, MODS, Xpert MTB/Rif, Diagnosis, Tuberculosis, HIV

## Abstract

**Background:**

Microscopic Observation Drug Susceptibility (MODS) and Xpert MTB/Rif (Xpert) are highly sensitive tests for diagnosis of pulmonary tuberculosis (PTB). This study evaluated the cost effectiveness of utilizing MODS versus Xpert for diagnosis of active pulmonary TB in HIV infected patients in Uganda.

**Methods:**

A decision analysis model comparing MODS versus Xpert for TB diagnosis was used. Costs were estimated by measuring and valuing relevant resources required to perform the MODS and Xpert tests. Diagnostic accuracy data of the tests were obtained from systematic reviews involving HIV infected patients. We calculated base values for unit costs and varied several assumptions to obtain the range estimates. Cost effectiveness was expressed as costs per TB patient diagnosed for each of the two diagnostic strategies. Base case analysis was performed using the base estimates for unit cost and diagnostic accuracy of the tests. Sensitivity analysis was performed using a range of value estimates for resources, prevalence, number of tests and diagnostic accuracy.

**Results:**

The unit cost of MODS was US$ 6.53 versus US$ 12.41 of Xpert. Consumables accounted for 59 % (US$ 3.84 of 6.53) of the unit cost for MODS and 84 % (US$10.37 of 12.41) of the unit cost for Xpert. The cost effectiveness ratio of the algorithm using MODS was US$ 34 per TB patient diagnosed compared to US$ 71 of the algorithm using Xpert. The algorithm using MODS was more cost-effective compared to the algorithm using Xpert for a wide range of different values of accuracy, cost and TB prevalence. The cost (threshold value), where the algorithm using Xpert was optimal over the algorithm using MODS was US$ 5.92.

**Conclusions:**

MODS versus Xpert was more cost-effective for the diagnosis of PTB among HIV patients in our setting. Efforts to scale-up MODS therefore need to be explored. However, since other non-economic factors may still favour the use of Xpert, the current cost of the Xpert cartridge still needs to be reduced further by more than half, in order to make it economically competitive with MODS.

## Background

In most low resource settings where Tuberculosis (TB) is huge problem, diagnosis conventionally relies on microscopy. However, TB microscopy has a sensitivity of only 40–60 % under field conditions, falling to as low as 20 % in the presence of HIV co-infection [1]. Two-thirds of HIV infected people live in sub-Saharan Africa, and 75 % of the global burden of HIV-associated TB is found in the region with limited health care resources [2]. With the launch of the Global Laboratory Initiative, strengthening and modernization of TB laboratories in low resource settings became a priority for global TB control, particularly in high HIV prevalence settings [3]. Consequently, since 2007, the array of diagnostics for TB has expanded tremendously and several of them have been endorsed by the World Health Organization (WHO) in such settings [4, 5].

The Xpert MTB/Rif test (Xpert) is an automated rapid molecular test with high sensitivity for simultaneous detection of pulmonary TB (PTB) and rifampicin resistance in a one off-test [6].

Xpert relies on real time polymerase chain reaction (PCR) to amplify a portion of Mycobacteria DNA. The steps involved in processing the sample, amplification and detection of the Mycobacterial DNA are automated. This enables reporting of test results in two-three hours [7].

A number of modelling studies in settings with high prevalence of TB-HIV co-infection, found Xpert was cost effective for diagnosis of PTB and reducing mortality in comparison to microscopy or conventional mycobacterial culture [8–11]. Thus, the WHO currently recommends Xpert as the primary diagnostic for HIV-associated TB as a replacement for TB microscopy [12]. Through the support of international donors and multilateral development assistance partners, the Xpert test has been rolled out on a large scale in several sub-Sahara African countries where TB and HIV co-infection is prevalent [13, 14]. However, the rollout of Xpert is faced with affordability and implementation challenges [15, 16]. There is also emerging evidence currently, that using Xpert in resource limited health-care settings may not be cost-effective because of its limited impact on patient mortality [17, 18].

The microscopic observation drug susceptibility (MODS) assay is an inexpensive test with high sensitivity for diagnosis of PTB in HIV infected patients [19], targeted for resource-limited settings [20, 21]. MODS is a liquid culture test, for simultaneous detection of TB and resistance to both rifampicin and isoniazid. MODS relies on two well-known properties of *Mycobacterium tuberculosis* (MTB): First, the rate of growth of TB bacilli in liquid medium is considerably higher than that on solid medium. Second, the morphology in liquid culture is characteristic and recognizable, consisting of so called “cord” like structures. By using an inverted light microscope to examine culture plates inoculated with sputum from patients with presumptive TB, MTB growth can be detected within 7–10 days, for both smear positive and negative samples, compared to conventional solid culture that takes 3–8 weeks [22, 23]. The MODS test has received increased attention in recent years and has been improved and standardized further for more widespread use [24, 25]. However, there is inadequate information about the full cost of the MODS procedure, including costs of materials, labour, laboratory equipment and overhead, which need to be properly evaluated.

The comparable diagnostic performance of the Xpert test with MODS and the urgent need of affordable tests for diagnosis of TB in HIV-infected patients, led us to perform this study in our setting in Uganda where HIV and TB are a high burden with an estimated incidence of 0.51 per 100 person year and 161 per 100,000 population respectively [26, 27]. The aim was to compare the cost-effectiveness of the utilizing the MODS test versus Xpert as primary tests for diagnosis of pulmonary tuberculosis (PTB) among patients infected with HIV. Our results could be useful for low income settings where implementation of the tests is planned or is already established.

## Methods

### Study population

The study population comprised adult HIV-infected patients older than 18 years, with presumptive active pulmonary TB. An HIV-infected patient could present with presumptive PTB regardless of whether they were on anti-retroviral treatment or not, CD4 count, HIV clinical stage or history of previous treatment for TB. An HIV patient was presumed to have active PTB if they had cough for two or more weeks with or without fever, night sweats, loss of weight, or blood stained sputum [18].

### Study setting

The diagnostic procedures were conducted in a TB research laboratory located within the campus of Mulago National referral Hospital in Kampala, Uganda. Sputum specimens were obtained from consecutively presenting patients to the Mulago Hospital HIV outpatient clinic and from patients admitted to the medical department of the hospital. HIV-infected adults presenting with symptoms and signs of PTB were enrolled on the basis of the WHO TB screening criteria. Symptomatic patients provided a spot and morning sputum in a universal sterile sputum container. At the laboratory, the two samples were pooled and examined using MODS and Xpert.

### Diagnostic procedures

All tests were performed by trained technicians. For the Xpert MTB/RIF assay, a sample reagent was added to the pooled sputum sample in a 2:1 ratio. The mixture was incubated at room temperature for 15–30 min and agitated manually. A total of 1 ml of the mixture was introduced into an Xpert MTB/RIF cartridge, which was then loaded into a GeneXpert instrument, where the subsequent steps of sample lysis, nucleic acid extraction, and amplification occurred automatically. The instrument generates the test report automatically within 3 h which is printed and signed by the technician.

The MODS test was performed in a 24-well tissue plate. The patient sputum was processed (digestion and decontamination) with NALC/NAOH 2 % method for 15 min, followed by homogenization. The homogenized sample was then centrifuged at 3000 X g (Allegra® X-12 series) for 15 min to prepare a sediment. The sediment was re-suspended with phosphate buffer (pH 6.8) to make 1–2 ml. The media for the MODS was prepared with 4.7 g Middlebrook 7H9 broth (Difco, Sparks, MD) and 2 ml glycerol in 900 ml of distilled water. This media was autoclaved at 121 °C for 10 min, cooled to 45 °C and enriched with 100 ml of Oleic, Albumin, Dextrose, catalase (OADC). A portion of the processed sample (100 μl) and Middlebrook 7H9 broth (800 μl) and of antibiotic mixture (100 μl) of polymyxin B, Amphotericin B, Nalidixic acid, Trimethoprim and Azlocillin (PANTA) were then transferred into wells giving a final volume of 1 ml/well. Two wells were used for each processed and quality control sample. For positive control, 100 μl of a suspension of H37Rv isolate 0.5 McFarland standard, was used. For negative control, 800 μl of Middlebrook 7H9 broth, 100 μl PANTA without sample was used. The tissue plates were sealed with tape and ziplock bags and incubated at 37 ^o^C. They were examined under an inverted light microscope at magnifications of X10 and X40 for cord formation.

### Model structure

**Fig. 1:**
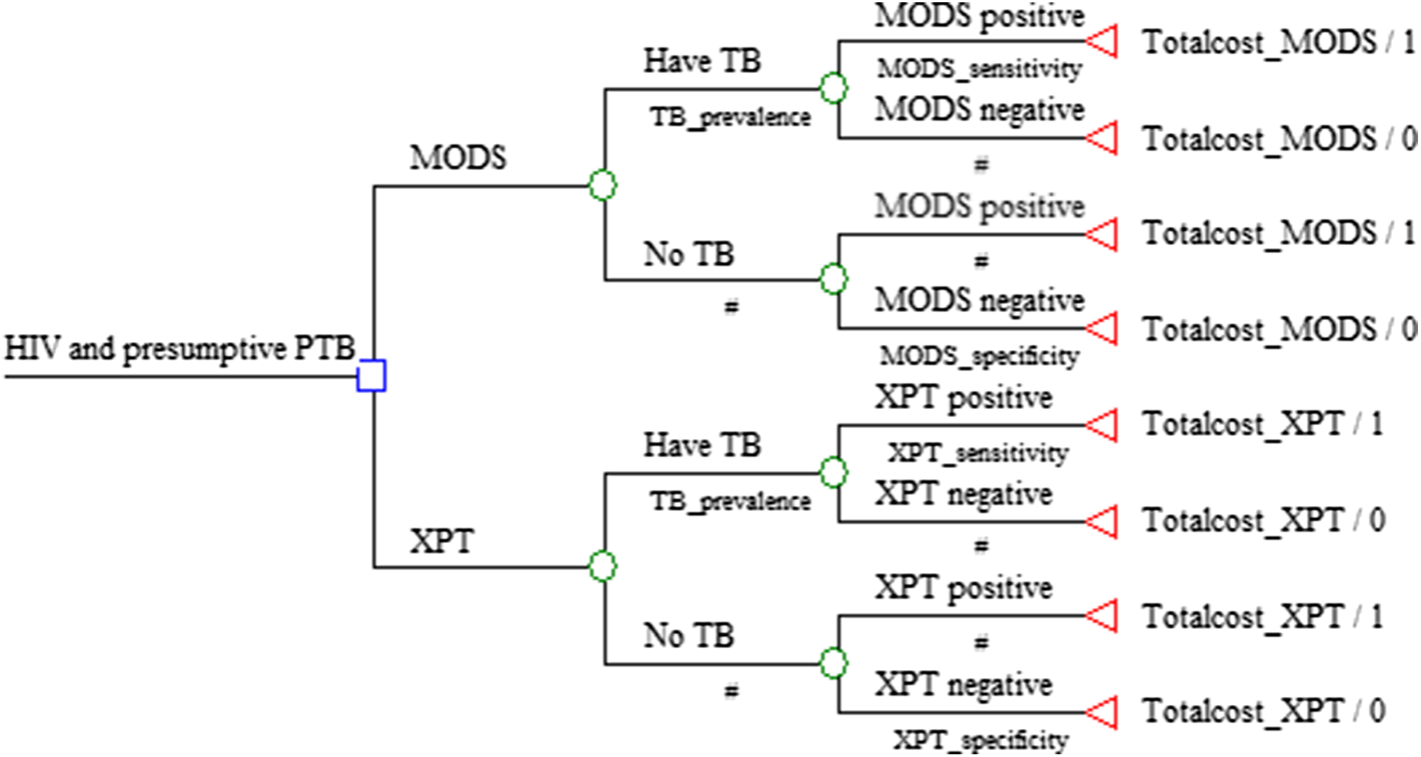
Decision analysis model for diagnosis of pulmonary tuberculosis in HIV patients using MODS or Xpert strategy

A decision-analysis model was constructed using TreeAge software (version 3.5) to compare the cost effectiveness of the MODS algorithm to the Xpert algorithm for diagnosis of TB (Fig. 1). The model involved 10,000 HIV patients with presumptive PTB. A positive MODS or Xpert test was either a true positive or a false negative based on the sensitivity of MODS or Xpert. A negative test was either a true negative or a false positive based on the specificity of MODS or Xpert.

### Model parameters

The data for diagnostic accuracy in the model were sourced from systematic reviews of studies on diagnostic accuracy of Xpert [28] and MODS [19, 20] among HIV infected patients. We used the pooled values from the systematic reviews as the base estimates for sensitivity and specificity of the tests, and the 95 % confidence interval values as the outer limits for diagnostic accuracy of the tests (Table 1).

**Table 1:** Model assumptions for TB diagnosis using MODS or Xpert

Model input	Base value	Min-Max	Reference
# Test sensitivity Xpert	0.79	0.70–0.86	[6]
# Test specificity Xpert	0.98	0.97–0.99	[6]
# Test sensitivity MODS	0.88	0.86–0.9	[19]
# Test specificity MODS	0.98	0.97–0.99	[19]
# TB prevalence	0.20	0.10–0.30	[49–52]

### Cost data

Estimates of the costs were made from the provider’s perspective (Table 2). The costs for the diagnostic procedure of each test were collected by identifying all the reagents required to perform the test and their quantities. These were assessed by reviewing the standard operating procedures (SOP) of the tests and observation of laboratory technicians during performance of the tests in the research laboratory in Mulago Hospital. We then computed the cost per test by applying a price per quantity of the resource used for the respective test. Prices of the reagents, equipment, calibration and training costs were obtained from laboratory invoices between 2010 and 2014. All costs were estimated in US$ based 2014 prices. The local costs were converted using the average exchange rate for 2014 of 2700 Uganda shillings for one US dollar. In the model we included some sunk costs, e g water, space and overheads, but since these costs were the same for the two alternatives, they were automatically cancelled out in the calculation. We also reviewed previous studies and guidelines to direct our data collection [8, 29–32]. We used standard tables of annualization and a discount rate of 3 % for the capital equipment. We made the following general assumptions to arrive at the unit costs for each test: With regard to equipment, the useful life of the centrifuge, incubator, autoclave, biosafety hood, inverted microscope were assumed to be 10 years and 100 % of their use was allocated to the MODS test. The useful life of a laboratory fridge was assumed to be 10 years and 20 % its use was allocated to the MODS test. The useful life of the GeneXpert machine was assumed to be 5 years and 100 % of its use was allocated to the Xpert test [33]. We used the concessional price of the GeneXpert machine and cartridges which is provided to resource poor settings. The useful life of digital pipettes was assumed to be 3 years and 100 % of their use was allocated to the MODS test. We assumed that both tests would require 25 M^2^ of work space and allocated the cost for space equally between the two tests. We allocated staff salary based on the time required to process the MODS test (2 h) and Xpert test (30 min). We assumed a maximum of 20 tests per day for MODS and 16 tests per day for Xpert and a total of 264 working days per year. We assumed MODS training required 22 days and Xpert 5 days and a refresher training for both tests by the laboratory staff every 3 years.

**Table 2:** Provider costs involved in the MODS and Xpert diagnostic procedure (US $)

	Method		
Component	MODS	Xpert	Source
Equipment Xpert test			
# Xpert MTB/Rif Machine 4 module and accessories	–	19900	Invoice
Equipment MODS test			
# Centrifuge (Beckmann Coulter x12R)	18000	–	Invoice
# Incubator (CO_2_)	19500	–	Invoice
# Autoclave	24500	–	Invoice
# Bio-safety cabinet (class 2)	11500	–	Invoice
# Inverted microscope	2700	–	Invoice
# Fridge	1300	–	Invoice
# Vortex	524	–	Invoice
# Pipettes (200ul-1 ml pipette)	218	–	Invoice
# Pipettes (50ul-200ul pipette)	218	–	Invoice
Consumables			
# Xpert cartridge & reagent kit	–	10	Invoice
# MODS culture media per year	245	–	Invoice
# MODS Culture plate	5	–	Invoice
# MODS digestion & decontamination reagents per year	416	–	Invoice
Staff			
# Annual salary for a laboratory technician	4000	4000	Invoice
# Training costs (5 days for Xpert, 22 for MODS)	990	225	Invoice
Overheads: Utilities, space			
# Utilities (water, power, stationary) per year	540	540	Invoice
# Space cost per M^2^ (25 M^2^ for either Xpert or MODS)	463	463	Invoice
Quality control			
# Xpert calibration cartridge per 2000 tests	–	450	Invoice
# MODS proficiency panels per year	940	–	Invoice

### Model outcomes

The model’s outcome measure were cost per TB patient diagnosed when the MODS test or the Xpert test were used for TB diagnosis in HIV infected individuals. We also derived incremental cost-effectiveness ratios (ICERs), expressed as US $ per TB patient diagnosed.

### Sensitivity analysis

We performed sensitivity analysis of our model based on adjustments of: (1) the diagnostic accuracy of the MODS and Xpert tests using the minimum and maximum values from the systematic reviews, (2) the useful life of the capital equipment of both tests between five and ten years, (3) the average number of tests performed per day between five and twenty, (4) different prices of the reagents for MODS and the Xpert cartridge, (5) the percentage allocated for shared equipment or staff time, (6) TB prevalence (10–30 %), corresponding to the most common values in this patient group from the studies in the systematic reviews.

### Data analysis

Cost data was entered and analyzed in Excel. The cost of equipment for each test was obtained by dividing the annualized cost of the equipment over the number of tests performed each year. The cost of consumables for each test was obtained by the dividing the gross cost of a given measure of each reagent over the average number of tests that can performed using that amount. The cost of quality control (QC) for a MODS test was obtained by dividing the total costs incurred for QC per year over the average number of MODS tests that can be performed each year. The cost of QC for Xpert was obtained by dividing the cost of the Xpert calibration cartridge over 2000 tests, which is the number recommended by the manufacturer when QC should be performed. Cost-effectiveness analysis was performed by in-putting the test probabilities and unit costs into the TreeAge software. A base case analysis was performed using the pooled estimates for diagnostic accuracy of the tests. Sensitivity analysis was performed by modifying the parameters in the model.

## Results

### Cost

The average cost for the MODS test was US$ 6.53 compared to US$ 12.41 for the Xpert test. Consumables (reagents and chemicals) accounted for 59 % of the cost for the MODS test while the Xpert cartridge with the reagent kit accounted for 84 % of the cost for the Xpert test (Table 3).

**Table 3:** Costs of MODS and Xpert by type of input, (2014$)

	Method, (% of total)	
Type of input	MODS	Xpert
Total (US$)	6.53	12.41
# Equipment	1.76, (27)	1.37, (11)
# Consumables	3.84, (59)	10.37, (84)
# Staff (salary and training)	0.46, (07)	0.15, (01)
# Quality control	0.18, (03)	0.23, (02)
# Overheads (utilities and space)	0.29, (04)	0.29, (02)

The effect of changes in the base-case assumptions on the unit cost of MODS and Xpert are summarized in Table 4. In the case of MODS, reducing the useful life of the capital equipment from ten to five years, increased the cost of the test moderately to US$ 8.04. Reducing the number of tests performed each day to five from twenty increased the cost of the test substantially to US$ 11.8. Increasing the price of reagents and chemicals by two-fold increased cost of the test minimally to US$ 7.8. Allocating 100 % of all shared equipment and staff time to MODS increased the cost for the test moderately to US$ 8.9.

**Table 4:** Effect of changes in the base-case assumptions on the unit cost of MODS and Xpert, (US $)

Type of test/parameter	Effect on cost	Increase/decrease (%)
MODS (base estimate US$ 6.53)		
-Reduce useful life of capital equipment from 10 to 5 years	8.04	+23
-Reduce number of tests to 5 each day	11.8	+ 81
-Double price of consumables	7.8	+ 19
-Allocate 100 % of shared equipment & staff time to MODS	8.9	+ 36
Xpert (base estimate US$ 12.41)		
-Increasing useful life of Xpert from 5 to 10 years	6.5	- 48
-Reduce number of tests to 5 each day	11.8	- 5
-Reduce price of cartridge by half	7.1	- 43
- Allocate 100 % of staff time to Xpert	13.2	+ 6

In the case of Xpert, increasing the useful life of the GeneXpert machine from five to ten years lowered the cost of the test substantially to US$ 6.5. Reducing the number of tests performed each day to five from sixteen lowered the cost of the test minimally to US$ 11.8. Reducing the cartridge price and reagent kit by two-fold lowered the cost of the test substantially to US$ 7.1. Allocating 100 % of staff time to Xpert raised the cost of the test minimally to US$13.2.

### Outcomes and Cost effectiveness

The MODS test generally detected more PTB patients by 11 % (range 5–23 %) compared to the Xpert test. In the base-case analysis, involving a cohort of 10,000 HIV patients with a PTB prevalence of 20 %, the algorithm using MODS would diagnose 1920 patients compared to 1740 patients by the algorithm using Xpert. The cost-effectiveness ratio of using MODS was US$ 34 per TB patient diagnosed compared to US$ 71 when using Xpert. The algorithm using MODS therefore detected more patients at lower costs, making it dominant over the algorithm using Xpert (Table 5).

**Table 5:** Cost-effectiveness of TB diagnosis using MODS or Xpert in a base-case analysis for a cohort of 10,000 HIV presumptive PTB patients

Strategy	Mean cost per test ($)	incremental cost per test ($)	Cases detected	Incremental cases detected	Cost effectiveness	ICER
MODS	6.53		1920		34	More cost effective
Xpert	12.41	(5.88)	1740	180	71	Dominated

### Sensitivity analysis

**Fig. 2:**
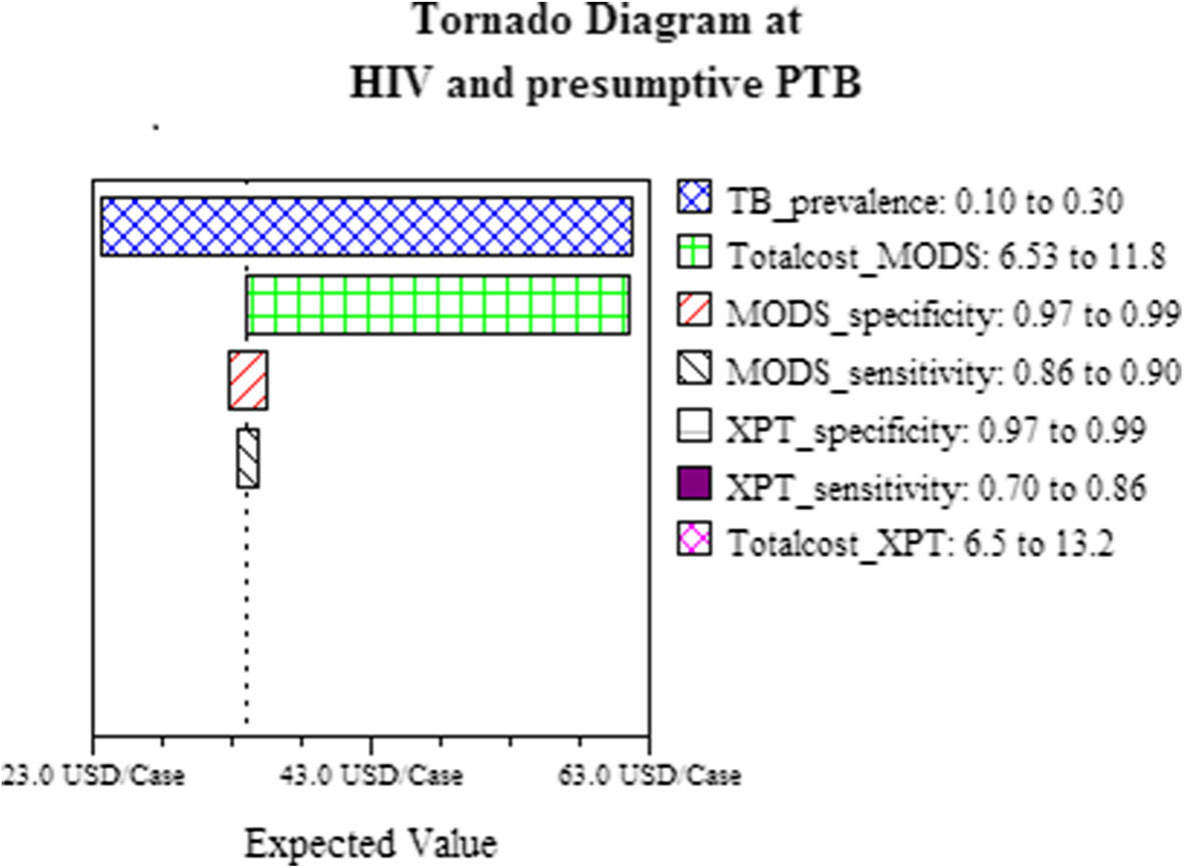
One-way sensitivity analysis comparing the influence of model parameters on cost effectiveness of MODS versus Xpert strategy for diagnosis of pulmonary tuberculosis in HIV patients. The x-axis is the cost per patient diagnosed. Each horizontal bar represents a parameter varied over the range indicated. Wider bars indicate larger differences in the cost per patient diagnosed by varying the parameter

In one-way sensitivity analyses, TB prevalence, followed by the cost of the MODS test had the most influence on results. The accuracy of the tests had the least influence (Fig. 2). However, the ratio of the total costs for diagnosis of TB patients using either the MODS or Xpert algorithm remained constant across variable prevalence situations (Table 6). The dominance of the algorithm using MODS was persistent across various values of PTB prevalence and for all the cost values of Xpert between 6.5–13.2 US$. The threshold value for cost, where using the Xpert algorithm would be optimal over the MODS algorithm was US $ 5.92.

**Table 6:** Expected diagnostic yield and costs of MODS versus Xpert strategy across variable prevalence situations for a cohort of 10,000 HIV presumptive PTB patients

Prevalence/Strategy	Total TB cases detected	Cost per case detected	Total cost for cases detected	Ratio total cost
TB prevalence (10 %)				
MODS	1060	62	65720	
Xpert	970	128	124160	1.89
TB prevalence (20 %)				
MODS	1920	34	65280	
Xpert	1740	71	123540	1.89
TB prevalence (30 %)				
MODS	2780	23	63940	
Xpert	2510	49	122990	1.92

## Discussion

We evaluated the cost-effectiveness of using an algorithm based on the MODS test versus Xpert test for diagnosis of Pulmonary Tuberculosis (PTB) in HIV patients from the perspective of the provider. The MODS algorithm was dominant over the Xpert algorithm despite adjustments in test accuracy, cost and TB prevalence.

Prevalence of PTB and the costs of the tests were the most influential parameters in our findings. However, while the prevalence of PTB had high influence on the cost per TB patient detected, it did not change the ratio of the total costs for the cases detected using either MODS or Xpert. Thus, in settings where prevalence of PTB in HIV patients is for example 30 % or more, implementation of the Xpert algorithm could be worthwhile despite the higher total costs incurred in comparison to MODS algorithm.

For both tests, the consumables had the most influence on the unit costs, although variation in the useful life of equipment and the average number of tests done each day also had substantial influence on the unit costs. The threshold for cost in order for Xpert to be optimal over MODS for diagnosis of PTB in our study population was about US$ 6. This value lies within the recommended US$ 4–6 for any new diagnostic to be placed at the microscopy-center level of the health care system [34]. This therefore would require further reduction of the currently subsidized price of the Xpert cartridge by more than half.

We did not consider data and costs of X-ray in our study. This is because we focused on new tests (MODS and Xpert) that provide a definitive (bacteriological) diagnosis of PTB which is important to ensure correct TB treatment. Although radiological tests such as X-ray have an important role in evaluating presumed PTB patients, their use often results in over diagnosis of PTB [35, 36]. They are therefore utilized primarily for diagnosis of extra-pulmonary TB and to assess presumed PTB patients for other etiologies of respiratory illness. In regard to cost-effectiveness analysis the costs of X-ray would cancel out one another since they would be the same in either the MODS or Xpert diagnostic algorithm.

A recurrent concern limiting the use of MODS is the total cost of the test arising from infrastructure requirements. While it is argued that roll out of Xpert requires minimal laboratory modifications, the costs involved in modifying available space to make it suitable for operating the Xpert test, and the costs for installation of some accessories like power inverters, air conditioners, have limited its placement at the microscopy-center level [37, 38]. Moreover, we found in our study that equipment and space accounted just over 30 % of the total cost of the MODS test. The MODS could therefore be a promising method for decentralizing sputum culture services up-to the microscopy-center level of the health care system. Besides, the test provides a platform for extended drug-susceptibility testing for drug resistant TB and can be assembled on site. Therefore, despite the low incidence of drug resistant TB among newly diagnosed patients in Uganda [39], MODS would offer rapid diagnosis of drug-resistant TB in a one-off test. On the other hand, presumed drug-resistant patients identified using the Xpert test require confirmation with culture. Thus additional costs are incurred in such situations. Our study therefore, could have under-estimated the benefits of implementing MODS in our setting.

Even though the MODS was the preferred alternative in our cost-effectiveness analysis, the Xpert has several advantages over the MODS test in regard to time to detection, biosafety, level of skills required to operate the test, labour intensity during performance of the test, and minimal variation in the test performance and quality assurance.

The MODS requires a median of seven days to detect growth in comparison to Xpert that provides results in 3 h allowing for same day detection and treatment. Xpert therefore has more potential than MODS to avert patient loss during the process of TB diagnosis. Xpert also has more potential to avert transmission of disease arising from early treatment upon detection of TB [40]. Unfortunately, although the turn-around-time of Xpert is short, in real life settings there is significant delay in getting the Xpert results and initiating TB treatment [41, 42] which counters these potential benefits.

Further, current Treatment algorithms suggest that all patients with positive Xpert results should immediately start anti-tuberculosis treatment. However, Xpert can detect DNA from both viable and nonviable TB bacilli. This presents a challenge that needs to be addressed as the Xpert test is rolled-out. This is important because despite the low likelihood of false Xpert positivity among new TB patients, false Xpert positivity among previously treated PTB patients may be common. [43, 44]. In such situations, clinicians may consider awaiting confirmatory testing using culture tests-which is a major advantage the MODS offers

A major advantage of utilization of Xpert is the limited concerns about biosafety during its use. On the other hand, biosafety is an important concern with the MODS and the test has so far therefore, been limited to referral or research TB culture laboratories. The risks about utilization of the MODS could however, be addressed by undertaking the procedures to perform the test inside a biosafety (Class 2) cabinet and having personal respiratory protection for laboratory staff such as N-95 masks. Further, since the MODS simply involves the inoculation of a sputum sample into a plate, after which the plate is sealed within a plastic bag and never again opened, the biosafety risks of the MODS test could be comparable to sputum smear microscopy as one study has shown previously [45].

MODS is more labour intensive and requires more skilled training to perform in comparison to Xpert. Recent innovations enabling automated interpretation of the test could make the labour and skills required to perform the test comparable to Xpert [46]. This could enable deployment of the tests to peripheral laboratories even more feasible. Still, there would be need to standardize the procedures for the test and set up quality assurance systems. Currently, the probability of invalid results from the MODS test requiring repeat testing, is comparable to Xpert but could be reduced further through these measures [38].

Cost-effectiveness analysis is not an evaluation of affordability. Thus the affordability of deploying the MODS or Xpert in relation to the current and future economic developments in several of the resource poor settings was not answered by our study. One study that has evaluated the cost and affordability of Xpert found that targeted use of the test would be affordable in the majority of high burden TB countries [47].

The study also did not compare the epidemiological and health system effects of using either MODS or Xpert for diagnosis of HIV associated TB. One study that evaluated the population effects of Xpert found that the test could substantially reduce the TB burden in a resource limited and HIV prevalent setting [11]. A similar study involving MODS is required given the dominance of MODS over Xpert. Based on modelling, MODS could have similar population effects with Xpert [48]. Our study assumed only a single diagnostic attempt during the patient’s disease course with no repeat diagnostic attempts. We also did not explore diagnostic attempts for multidrug-resistant tuberculosis using either MODS or Xpert.

## Conclusions

The algorithm using MODS was more cost-effective compared to the algorithm using Xpert for the diagnosis of TB among HIV patients in our setting. Efforts to scale-up MODS therefore need to be explored. However, other non-economic factors may still favour the use of Xpert in our setting or other similar settings. But the current cost of the Xpert test, with subsidies, needs to be reduced further by more than half to make it economically competitive with MODS.

## Abbreviations

HIV: Human immune deficiency virus
M.TB: Mycobacterium tuberculosis
MODS: Microscopic observation drug susceptibility test
PTB: Pulmonary tuberculosis
QC: Quality control
SOP: Standard operating procedures
TB: Tuberculosis
Xpert/XPT: Xpert MTB/Rif test
